# Form and Temporal Integration in the Perception of Simple Glass Patterns

**DOI:** 10.3390/vision9030069

**Published:** 2025-08-04

**Authors:** Rita Donato, Michele Vicovaro, Massimo Nucci, Marco Roccato, Gianluca Campana, Andrea Pavan

**Affiliations:** 1 Department of General Psychology, University of Padova, Via Venezia 8, 35131 Padova, Italy; michele.vicovaro@unipd.it (M.V.); massimo.nucci@unipd.it (M.N.); marco.roccato@studenti.unipd.it (M.R.); gianluca.campana@unipd.it (G.C.); 2 Human Inspired Technology Research Centre, University of Padova, Via Luzzati 4, 35121 Padova, Italy; 3 Department of Psychology, University of Bologna, Viale Berti Pichat 5, 40127 Bologna, Italy; andrea.pavan2@unibo.it

**Keywords:** translational Glass patterns, form–motion processing, form summation, temporal integration

## Abstract

This study presents a reanalysis of existing data to clarify how the visual system processes simple dynamic Glass patterns (GPs), with a particular focus on translational configurations. By combining datasets from previous studies, we apply a mixed-effects modeling approach—which offers advantages over the statistical methods used in previous studies—to investigate the contributions of pattern update rate and number of unique frames to perceptual sensitivity. Our findings indicate that the number of unique frames is the most robust predictor of discrimination thresholds, supporting the idea that the visual system integrates global form information across multiple frames—a process consistent with spatiotemporal summation. In contrast, the pattern update rate showed a weaker, though statistically significant, effect. This suggests that faster updates help preserve temporal consistency between frames, facilitating global form extraction. These results align with previous observations on complex dynamic GPs, where discrimination thresholds decrease with more unique frames, suggesting that the summation of form signals across time plays a key role in form–motion perception. By adopting a mixed-effects modeling approach, our reanalysis provides new insights into the mechanisms underlying global form perception in dynamic GPs.

## 1. Introduction

Glass patterns (GPs), introduced by Glass [1], are widely used in vision neuroscience and psychophysics to investigate the mechanisms underlying form and motion processing in the visual system [2,3,4,5,6,7,8,9,10,11]. GPs consist of pairs of dots called *dipoles*, arranged to convey different global forms such as translational, circular, radial, or spiral structures. Translational GPs can be classified as simple patterns because the dipoles exhibit a uniform orientation, such as vertical, horizontal, or tilted at various angles. In contrast, circular, radial, or spiral GPs are classified as complex patterns because the dipoles forming these global shapes have different orientations; consequently, the visual system must perform more complex computations to perceive the global structure. At the neural level, early visual areas (e.g., V1/V2) are known to encode local orientation and position information [12], while higher-level occipitotemporal regions such as V4 and the lateral occipital areas are more involved in integrating global form [13,14,15]. Functional imaging and physiological evidence suggest a hierarchical and interactive processing system, in which global shape perception arises from the pooling and integration of local cues along both feedforward and feedback pathways [16]. Moreover, GPs can be either static or dynamic. Static GPs consist of a fixed arrangement of dipoles that form a global structure without temporal variation; the dipoles have specific orientation. Static GPs are primarily associated with spatial integration, whereby the brain interprets the spatial relationships between dipoles to detect a global shape [17,18]. In contrast, dynamic GPs are composed of sequences of unique, independent frames containing static GPs that are presented over time, with each frame updated at a certain rate. While the dipoles within these frames vary randomly in position, their orientation and inter-dot distance remain constant across frames. This rapid succession of frames induces the perception of motion along the orientation axis of the pattern, despite the absence of dipole-to-dipole correspondence between frames. As a result, no coherent motion is present, and the perceived motion is directionally ambiguous [19,20,21,22]. Due to these characteristics, dynamic GPs involve both spatial and temporal processing, as the visual system must integrate the spatial configuration of dipoles across multiple frames and extract the global form despite the random repositioning of dipoles from one frame to the next. Thus, dynamic GPs have been particularly useful for studying how the brain processes temporal and motion information, as they engage visual cortical areas related to motion perception, such as the motion complex hMT+ [3,23,24,25,26,27,28].

Based on their intrinsic features, dynamic GPs challenge the visual system by requiring a synthesis of form from temporally distributed and spatially irregular dipoles, thus engaging mechanisms of spatiotemporal integration across successive frames [29]. Studies using magnetoencephalography (MEG) and functional magnetic resonance imaging (fMRI) have revealed that such integration is supported not only by ventral stream structures but also by the dorsal stream [3,16], indicating reciprocal interactions between these two visual pathways. The dorsal pathway, traditionally associated with motion and spatial localization, also participates in form perception when temporal continuity must be inferred. The integration of form and motion in dynamic GPs also challenges classical models of modular processing [30,31]. Rather than reflecting a strict segregation between the ventral (“what”) stream, specialized for form recognition, and the dorsal (“where/how”) stream, specialized for motion and spatial analysis, recent evidence supports a model of distributed, interactive processing. Experimental studies with dynamic GPs suggest that shared and overlapping cortical mechanisms contribute to both form-from-motion and motion-from-form perception [32,33]. This interplay is further supported by evidence of cue invariance in hMT+, which responds to both real directional motion and illusory, non-directional motion generated by form cues in dynamic GPs [3]. In parallel, dorsal areas appear to support global form processing when visual cues unfold over time, as in dynamic GPs [16]. These findings highlight that perceptual organization involves cross-talk between motion- and form-sensitive regions, and that spatiotemporal coherence may arise from bidirectional interactions across the visual hierarchy.

In addition, the perception of dynamic GPs requires the segmentation of visual inputs that change over time into coherent form representations [18]. Since the dipoles change position across frames, the visual system cannot rely on fixed spatial relationships. Instead, it must group orientation cues that are spatially misaligned but follow the same global rule. In simple translational patterns, this rule corresponds to a single, constant orientation shared across all dipoles and frames. In complex patterns (e.g., circular, radial, or spiral), the orientations vary across space according to a specific geometric transformation. In these cases, what links the dipoles across frames is not identical orientation, but consistency with the same underlying transformation that defines the global shape. This form of temporal segmentation likely engages both early visual areas, which extract local orientation and contrast information [12,34], and higher-order regions, such as the lateral occipital cortex and hMT+, which contribute to global form integration across time [3,13]. Crucially, top-down feedback mechanisms are thought to stabilize these percepts by refining noisy or ambiguous input and supporting perceptual continuity [14,35]. Such mechanisms play a fundamental role when incoming sensory signals are fragmented or unstable, as in dynamic GPs. Through recurrent feedback from higher-level visual areas, the brain can iteratively reprocess early sensory representations, helping to resolve uncertainty and reinforce the perception of a coherent global structure [3]. Rather than being driven solely by feedforward inputs, perception in this context emerges from a dynamic interaction in which feedback loops shape and constrain the perception of the stimulus over time [35]. This recurrent process enables the visual system to maintain perceptual stability across rapidly changing frames, compensating for the lack of spatial continuity by enhancing temporally consistent features. Therefore, segmentation in dynamic GPs reflects a flexible and adaptive strategy, where bottom-up and top-down signals interact continuously to achieve perceptual coherence despite the inherent ambiguity caused by the lack of dipole-to-dipole correspondence and the spatial displacement of dipoles across successive frames [23].

Previous research has extensively examined the mechanisms underlying the perception of dynamic GPs [23]. For example, Day and Palomares [36] demonstrated that increasing the temporal frequency of dynamic GPs leads to a linear decrease in detection thresholds. They interpreted this effect within the framework of the *motion streak model* [5,24], which suggests that rapidly moving objects leave a blurred trail due to temporal integration, providing additional orientation cues that aid and improve motion direction discrimination [37,38,39]. However, while the authors emphasized the role of temporal frequency, their findings left open the question of whether improved sensitivity to dynamic GPs was solely due to temporal mechanisms or also to multiple global form signals. Inspired by this earlier work, Nankoo et al. [40] addressed this question. Specifically, they investigated whether dynamic GPs are more easily perceived than static GPs due to repeated exposure to global form signals or due to the higher pattern update rate at which frames are presented. Their findings suggested that the key factor was the number of unique frames, highlighting the importance of the cumulative form information provided by each successive frame. Building on these studies, Donato et al. [41] explored whether similar summation mechanisms apply not only to translational GPs but also to complex GPs, specifically circular dynamic GPs. By manipulating the number of unique frames and the update rate of frame presentation using the same method as Nankoo et al. [40], they found evidence that both factors equally influence detection thresholds. Further extending this research, Roccato et al. [42] examined how these mechanisms operate in different types of complex GPs, including circular, radial, and spiral patterns. The results indicated that circular GPs are easier to detect than radial and spiral ones, as they show lower discrimination thresholds. Moreover, in partial contrast with Donato et al. [41], they found that while increasing the number of unique frames significantly reduced coherence thresholds, variations in update rate did not. This suggests that form information from dipoles distributed across different frames plays a primary role in processing complex GPs.

The present study is based on these previous investigations but focuses exclusively on translational GPs. By reanalyzing data from Nankoo et al. [40] and Donato et al. [41], this study aims to further clarify how the visual system integrates form and motion information in the perception of dynamic translational GPs and to resolve existing partial inconsistencies in the literature regarding the detection of dynamic GPs. Additionally, by applying a mixed-model approach similar to that used by Roccato et al. [42], this research ensures continuity in the methodological framework. The use of linear mixed-effects models (LMMs) represents an advancement over the statistical approaches employed in previous studies. Specifically, Nankoo et al. [40] relied on partial correlation analyses computed within individual participants and then aggregated across the sample. While this approach allows for the control of covariates at the individual level, it does not account for inter-individual variability and assumes independence between predictors, which is problematic in this context, as the number of unique frames and the pattern update rate are partially collinear. Indeed, certain combinations of values occur only in specific experimental conditions. Similarly, Donato et al. [41] used repeated-measures ANOVA with the implicit assumption that the predictors varied independently, and that variance was homogeneously distributed across conditions. These assumptions are unlikely to hold in a design where predictors are not fully orthogonal. In contrast, LMMs allow for both predictors to be modeled simultaneously, taking their correlation into account, and can include random effects to account for subject-level variability without the need to average or transform individual estimates. This approach retains the full structure of the dataset and improves statistical power and generalizability.

## 2. Method

### 2.1. Stimuli and Design

**Figure 1:**
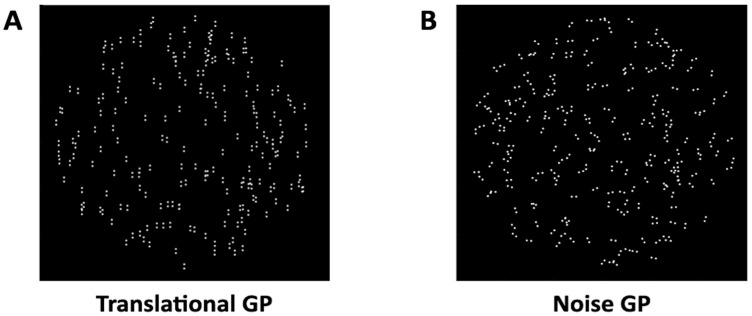
(**A**) Example of a translational GP with dipoles oriented vertically, illustrating coherent global structure. (**B**) Example of a noise GP, where dipoles are randomly oriented and lack global coherence. These images are for illustrative purposes only; dipole size and number have been adjusted to enhance the visual explanation of translational and noise GPs.

This study analyzes data from twenty-nine adult participants with normal or corrected-to-normal vision, who took part in two previously published experiments [40,41]. The visual stimuli were translational and noise GPs (Figure 1). GPs consisted of 2146 white dipoles (density: 6%) displayed against a black background. The separation between dots was 0.25 degrees, with each dot measuring 0.04 degrees in diameter. These GPs appeared within a circular window inside an annulus with a maximum radius of 5.35 degrees (total diameter: 10.7 degrees).

Static GPs were formed from a single unique frame, whereas dynamic GPs consisted of multiple independent frames shown in rapid succession, each lasting 0.0167 s. The stimulus was presented for a total duration of 0.2 s. Table 1 [40] details the number of unique frames and pattern update rates for each of the nine experimental conditions. In each condition, frame sequences were varied to manipulate the temporal characteristics of the GP. All participants completed all nine conditions.

Discrimination thresholds were estimated using different adaptive procedures in the two studies. Nankoo et al. [40] used the QUEST staircase method [43], an adaptive Bayesian procedure that updates a posterior probability distribution of the threshold on each trial and selects the next stimulus accordingly. This method fits a psychometric function to the accumulated data and derives the threshold estimate from the fitted curve.

In contrast, Donato et al. [41] employed the Updated Maximum-Likelihood (UML) method [44,45], which selects stimuli to maximize expected information gain when estimating the parameters of a cumulative Gaussian psychometric function. Their implementation used a 1-up/3-down rule and explicitly modeled lapse rate and slope, with thresholds defined as the coherence level yielding 79% correct responses.

Despite these methodological differences, we do not believe they are the primary cause of the diverging conclusions reached in the two studies. This assumption is formally tested in our analysis by including the variable ‘study’ as a fixed effect in the statistical model, allowing us to assess whether outcome differences could plausibly be attributed to procedural variation.

### 2.2. Procedure

In Nankoo et al. [40], participants were shown a translational GP with varying numbers of unique frames and pattern update rate. The unique frames were presented in one of two formats: alternating or blocked. In the alternating sequence, the unique frames were presented in a repeating order, alternating between them across the 12 frames of each stimulus. In the blocked sequence, the unique frames were presented consecutively in blocks, where one frame was shown multiple times before switching to the next frame. The pattern update rate varied between 5 Hz and 60 Hz. Notably, in the first condition, the 0.2 s presentation time resulted in an update rate of 5 Hz, leading to the perception of static patterns. Participants completed a two-interval forced-choice (2IFC) task to detect a translational GP, identifying whether the signal-containing stimulus appeared in the first or second interval. The coherence of the stimulus was defined as the percentage of non-randomly oriented dipoles. Donato et al. [41] replicated the study by Nankoo et al. [40].

## 3. Results

All statistical analyses were conducted using R (v4.1.3) [46] in RStudio (v2024.04.2+764). The script with the complete statistical analyses is available online on OSF (https://osf.io/azynh/?view_only=a259e898898c4a6fa1bee3014defef5c, accessed on 30 July 2025). The dataset provided on OSF includes only the data from Donato et al. [41], for which we received permission to share. The data from the second study [40] have been excluded from the OSF repository in accordance with data sharing agreements. Access to the data may be obtained by contacting the authors of Nankoo et al. [40].

**Figure 2:**
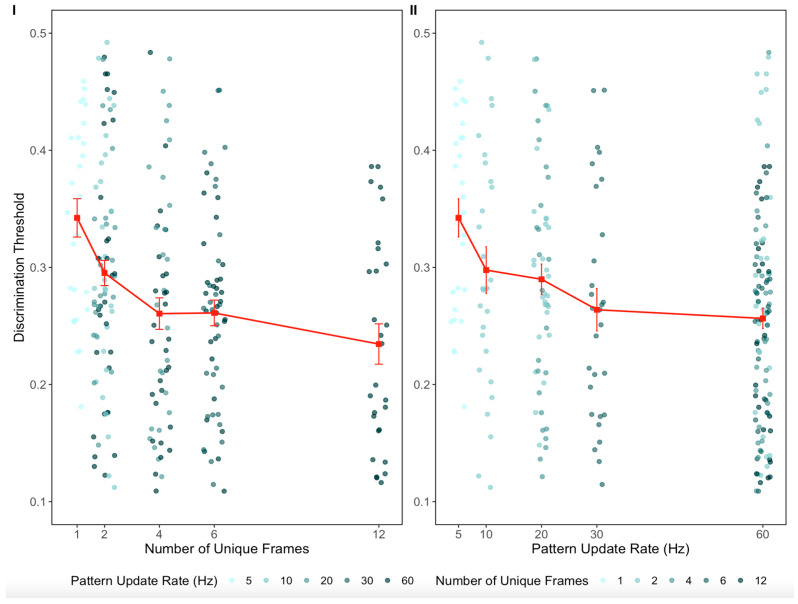
(**I**) Discrimination threshold as a function of the number of unique frames. (**II**) Discrimination threshold as a function of the pattern update rate. Both plots are based on raw data.

A preliminary analysis on log-transformed coherence thresholds showed that the study variable (two levels: Nankoo et al. [40] and Donato et al. [41]) had no main effect on discrimination thresholds and did not modulate the relationship between each predictor, i.e., number of unique frames (χ^2^(2) = 0.85, *p* = 0.65) and pattern update rate (χ^2^(2) = 1.19, *p* = 0.55) (see the OSF link, accessed on 30 July 2025). The aggregated untransformed data from the two studies, as a function of pattern update rate and number of unique frames, are shown in Figure 2.

Discrimination thresholds were log-transformed to linearize their exponential relationship with the predictors. Although this transformation introduced slight negative skewness, it yielded better linearity than both square root and Box–Cox transformations. Moreover, a Shapiro–Wilk test found that residuals were normally distributed (*p* ≥ 0.5).

Log-transformed thresholds were analyzed using linear mixed-effects models (*lme4* package in RStudio v2024.04.2+764) [47]. Five models of increasing complexity in the fixed part were fit: a null model; models with one predictor (either pattern update rate or number of unique frames); a model with both predictors without interaction; and a model with both predictors including the interaction. The random part initially included by-subject random intercepts to account for baseline variability (see the OSF link, accessed on 30 July 2025).

**Figure 3:**
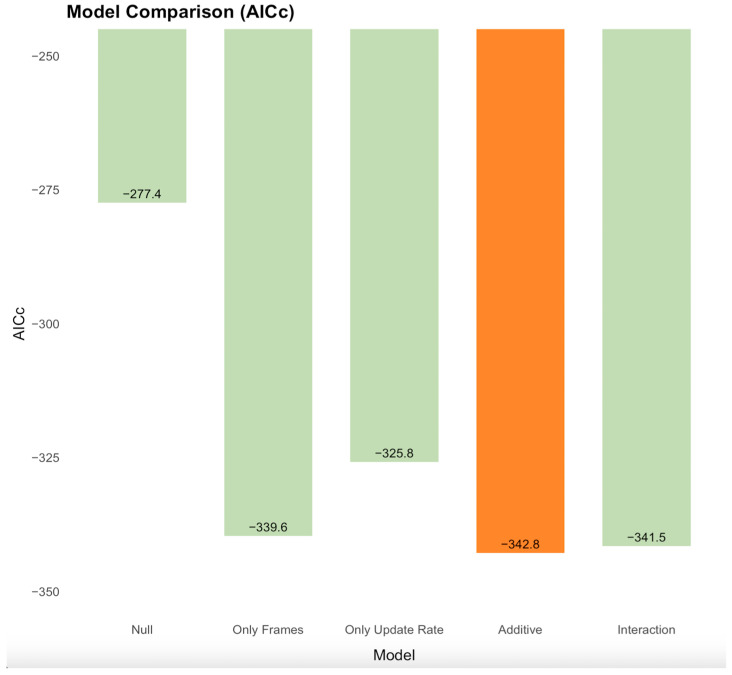
Comparison of model performance using corrected Akaike Information Criterion (AICc) values for five mixed-effects models predicting log-transformed thresholds. Lower AICc values indicate better model performance. The models are ordered on the x-axis as null (intercept only), only frames, only update rate, additive (main effects of frames and update rate), and interaction. The best-fitting additive model is highlighted in bright orange, while the other models are shown in light green.

Log-likelihood ratio tests indicated that the additive model (AICc weight = 0.587; marginal *R*^2^ = 0.112) provided a significantly better fit than the model with only pattern update rate (AICc weight < 0.001; *R*^2^ = 0.085; *χ*^2^(1) = 19.14, *p* < 0.001) and the model with only number of unique frames (AICc weight = 0.118; *R*^2^ = 0.105; *χ*^2^(1) = 5.28, *p* = 0.022). The interaction model did not significantly improve the fit (AICc weight = 0.295; *R*^2^ = 0.113; *χ*^2^(1) = 0.72, *p* = 0.40). Including by-subject random slopes for the predictors did not significantly improve model fit, suggesting negligible inter-individual variability in predictor effects. Figure 3 shows the performance comparison between the five mixed-effects models using corrected Akaike Information Criterion (AICc) values, highlighting the superior performance of the additive model.

Estimated fixed-effect parameters were *b* = −0.134 for number of unique frames (SE = 0.030; *t*(230.0) = −4.45; *p* < 0.001) and *b* = −0.058 for pattern update rate (SE = 0.025; *t*(230.0) = −2.30; *p* = 0.022). Although the effect of pattern update rate was statistically significant, it accounted for approximately 20% less of the explained variance than the effect of number of unique frames.

Further analyses conducted separately at two, four, and six unique frames (i.e., 10 vs. 20 vs. 60 Hz at two frames; 20 vs. 60 Hz at four frames; 30 vs. 60 Hz at six frames) revealed no statistically significant effect of the pattern update rate on discrimination thresholds in any condition. Thus, the weak effect of the pattern update rate emerges only when data are pooled across all levels of unique frames. In contrast, discrimination thresholds decreased significantly with the number of unique frames at 20 Hz (two vs. four frames) and at 60 Hz (two vs. four vs. six frames), indicating that the number of unique frames has a robust and consistent effect across different levels of pattern update rate (see the OSF link, accessed on 30 July 2025).

## 4. Discussion

This study set out to clarify how the visual system processes simple dynamic GPs, focusing on the relative influence of the number of unique frames and the pattern update rate on perceptual processing. To this end, we reanalyzed data from two independent experiments within a unified framework based on linear mixed-effects models (LMMs). This approach allowed us to jointly account for individual differences and correlations among predictors—factors that previous analyses had not fully addressed. Importantly, our modeling strategy builds on recent work by Roccato et al. [42], who used LMMs to disentangle stimulus-driven and observer-specific effects in tasks involving complex dynamic GPs with varying temporal structure. In their study, LMMs helped separate the contributions of frame sequence and participant-level variability to discrimination thresholds. Here, we extend their methodology to a different class of stimuli: simple translational dynamic GPs. By pooling data from the two studies, we substantially increased sample size and statistical power, allowing for a more reliable assessment of temporal influences on perceptual performance. Although both studies employed identical stimulus conditions and task designs, they differed in their psychophysical procedures: Nankoo et al. [40] used the QUEST adaptive staircase method, whereas Donato et al. [41] applied the UML method, which explicitly models lapse rates and psychometric slopes. To control for these procedural differences, our statistical model included ‘study’ as a fixed effect, thereby formally testing whether such methodological variance influenced the outcomes.

### 4.1. The Role of Unique Frames in Global Form Perception

Our results suggest that the number of unique frames is the most reliable predictor of the discrimination threshold. This aligns with previous research, such as the studies of Nankoo et al. [40] and Roccato et al. [42], which have emphasized the importance of global form cues in dynamic GPs. In their studies, the integration of form information over successive frames was found to enhance the discrimination/detection of global form in GPs. The integration of global form cues across frames likely leads to improved perceptual sensitivity, as the visual system is able to integrate diverse visuospatial information over time.

The importance of the number of unique frames forming dynamic GPs can be better understood through the concept of spatiotemporal summation. Spatiotemporal summation refers to the visual system’s ability to integrate spatially sparse form information within and across multiple frames of a stimulus over time, thereby improving the perceptual sensitivity to the overall form [42]. Unlike static GPs, which are characterized by only a single frame, dynamic GPs involve the integration of form information contained in the dipoles’ orientation not just within but also across multiple frames, enhancing perceptual sensitivity. Our results suggest that the increased sensitivity in dynamic GPs is primarily driven by the spatiotemporal summation of global form signals across different frames, rather than the rate at which the pattern is updated.

It is also worth noting that psychophysical and modeling work by Schmidtmann et al. [48] and Kingdom et al. [49] has challenged the traditional view that spatial integration in GPs relies on simple linear summation of local cues. By systematically varying the “signal area”—that is, the proportion of the pattern containing oriented Gabor/dipole elements—the authors showed that detection of concentric and other orientation-defined textures is best explained by probability summation within signal detection theory. Crucially, they found that the slope of the psychometric function decreases as the signal area increases, a hallmark of probability summation and inconsistent with linear integration. These works highlight that the complexity of global form integration in Glass patterns lies primarily in the underlying computational principle, rather than in the specific processing stages or brain regions involved. Although their work focuses on static (not dynamic) GPs, the finding that integration is governed by probability rather than strict linearity aligns with our results, which emphasize the importance of cumulative form information across both space and time in dynamic GPs.

Neuroimaging research by McCarthy et al. [33] provides further insight into the mechanisms underlying temporal integration of visual information. Their study showed that coherent shape percepts can emerge from stimuli in which simple visual elements—specifically, Gaussian blobs—are presented sequentially over time at different spatial positions, generating the perception of apparent motion. Across two experiments, they demonstrated that the orientation of these local elements can modulate the perceived speed of motion and, in turn, influence the perception of global object shape. They identified two complementary neural processes: spatiotemporal form integration (STFI), which enables the visual system to accumulate and maintain fragmented shape information across time, and position updating, which supports the alignment of spatially distributed elements along a coherent motion trajectory. STFI was found to involve early visual areas such as V2, as well as higher-level regions including LOC and V4v, while position updating additionally recruited motion-sensitive areas such as KO and hMT+, reflecting the integration of form and motion even in the absence of continuous physical displacement. Based on these findings, we can infer that the improved perceptual sensitivity in dynamic GPs is due to the greater variation between frames and may rely on similar neural computations, involving both the sustained integration of form information and the dynamic updating of spatial position over time.

This process of form integration over time is similar to what is observed in directional motion perception, where the brain integrates information over time to create a coherent perception of motion [50,51]. The ability to integrate visual information across frames in dynamic GPs engages neural processes that involve pooling of form information, potentially involving visual processing areas, such as V3a [16], the lateral occipital complex (LOC), which is known to play a key role in object recognition and form processing, and the motion complex hMT+ [3,25,52].

### 4.2. The Role of Pattern Update Rate

In line with the findings of Day and Palomares [36], who proposed that sensitivity to dynamic GPs improves with higher temporal frequencies due to the contribution of motion streak mechanisms, our results revealed a weak but statistically significant effect of the pattern update rate on discrimination thresholds. Although dynamic GPs do not exhibit continuous motion trajectories, rapidly refreshed dipoles may generate orientation signals akin to motion streaks. Therefore, the observed effect of pattern update rate may be partially attributed to motion streaks generated by rapidly refreshed dipoles [21,38,39]. A *motion streak* is a visual trace that extends along the axis of an object’s motion, analogous to speed lines drawn behind cartoon characters to indicate movement. These motion streaks provide orientation cues signaled by neurons in the primary visual cortex [37], which help integrate form and motion information to enhance perception of object trajectory. Geisler [37] argued that these streaks can assist in discriminating motion trajectories, particularly for faster stimuli where conventional motion detectors become less reliable. Dipoles’ orientations in GPs can simulate such motion streaks, influencing judgments about the illusory global motion direction. Specifically, Burr and Ross [38] demonstrated that when dipole orientations deviate slightly (within approximately 20°) from the actual motion axis, the accuracy of motion direction judgments decreases, indicating that these orientation signals may act as misleading cues. This idea was further extended by Krekelberg et al. [4], who showed that the perceived motion direction is a weighted average between the global form composed of local dipole orientations and the physical motion direction, with significant attraction of perceived motion toward dipole orientation at small conflict angles (10–45°). In addition, Pavan et al. [39] further demonstrated that orientation information in moving GPs can exert a strong influence on perceived motion direction, and that motion streaks arising from the dynamic integration of dipoles can dominate over orientation cues, especially when stimulus parameters favor streak formation. These findings highlight the asymmetrical interaction between form and motion signals and provide direct psychophysical evidence of their integration in the human visual system. Neural recordings in macaque visual areas MT and MST supported these behavioral effects, highlighting the interaction between motion and form processing [4].

However, it is also possible that other factors, such as the integration of global form cues across multiple frames, play a more significant role in determining perceptual sensitivity. These findings are in line with previous studies by Nankoo et al. [40] and Donato et al. [41], but partially diverge from the results reported by Roccato et al. [42], who focused on complex GPs and did not observe any significant effect of pattern update rate. This discrepancy may reflect differences in how simple and complex GPs are processed. In complex GPs, the varying orientations and spatial configurations might reduce the effectiveness of motion streaks, leading to a diminished impact of pattern update rate.

Furthermore, the plateau observed in discrimination performance at around four unique frames and a pattern update frequency near 20–30 Hz likely reflects inherent temporal limitations of the human visual system, particularly those associated with visual persistence and temporal integration. Visual persistence refers to the continued influence of a stimulus on perception for tens of milliseconds after its physical offset, typically lasting 30–50 milliseconds—corresponding to temporal frequencies of approximately 20–30 Hz. This suggests that increasing the update rate beyond this range may not further enhance perceptual discrimination, as successive frames begin to fall within the same integration window, resulting in perceptual overlap. Supporting this interpretation, Pavan et al. [20] found that the tilt after-effect elicited by translational GPs peaks around 30 Hz, indicating that orientation-selective neural populations are especially sensitive to temporal changes around this frequency. Together, these findings suggest that temporal integration and visual persistence impose a functional ceiling on the perceptual benefits of higher update rates.

### 4.3. Future Directions

Although no new data were collected in this study, the present reanalysis highlights several avenues for future experimental research. The robust effect of the number of unique frames on discrimination thresholds suggests that the visual system relies heavily on the spatiotemporal summation of global form cues. To further investigate this mechanism, future studies could explore how visual sensitivity is shaped by specific temporal and spatial manipulations. For example, varying dot spacing or systematically combining different pattern update rates with numbers of unique frames could clarify the interplay between temporal dynamics and global form processing. This approach, in combination with neuroimaging techniques, might also shed light on how the visual system transitions from local to global processing stages—i.e., how early feedforward mechanisms integrate local dipole orientations before feedback loops refine global structure perception. Mapping this perceptual space in greater detail would provide a more comprehensive understanding of how form, motion, and temporal integration interact.

Moreover, our results revealed a weak but statistically significant effect of the pattern update rate in simple translational GPs—a finding that contrasts with the lack of such an effect in the study by Roccato et al. [42] using complex GPs. This discrepancy suggests that different classes of GPs may engage distinct perceptual or neural mechanisms. One possibility is that, in simple translational GPs, the consistent dipole orientation across the display creates a stronger impression of illusory global motion when frames are updated rapidly. This illusory motion signal might increase the visual system’s sensitivity to temporal structure, even in the absence of actual directional motion. Such findings raise questions about the integration of form from motion: does the illusory motion perceived in fast-updating simple GPs reinforce form coherence, or is the reverse true—does the extraction of a stable form scaffold the illusory motion percept? This bidirectional interaction could reflect dynamic feedback between dorsal and ventral visual streams, a hypothesis that could be tested using paradigms employing psychophysical motion coherence task, neuroimaging techniques such as fMRI and electroencephalography (EEG). Additionally, the temporal structure of GPs may influence the grouping of dipoles into coherent percepts, especially under conditions of limited frame presentation. Future work could investigate how temporal predictability modulates the efficiency or dynamics of these integrative processes.

Finally, understanding how motion signals emerge from form features—and vice versa—could provide key insights into the hierarchical and interactive nature of visual processing. For instance, exploring how form and motion information are integrated or dissociated in brain areas such as hMT+, V3A, or LOC would clarify whether the integration of motion from form varies depending on stimulus characteristics or task-related factors. In this sense, GPs offer a valuable tool to probe the boundaries between form and motion systems, and how spatiotemporal information is flexibly routed and recombined across cortical hierarchies.

## 5. Conclusions

Our findings underscore the role of the number of unique frames in determining the discrimination threshold for dynamic GPs. On the other hand, the pattern update rate exhibited a statistically significant but weaker effect. These results highlights the importance of global form integration over time and suggests that simple translational GPs may engage different perceptual mechanisms compared to complex configurations, which might rely less on temporal dynamics.

## Figures and Tables

**Table 1:** The experimental conditions used in both Nankoo et al. [40] and Donato et al. [41]. The temporal parameters include the pattern update rate (in Hz), as well as the sequence and number of unique frames. Unique frames are represented by letters in the second column, with each letter corresponding to a distinct frame.

Condition	Sequence of Unique Frames	Number of Unique Frames	Pattern Update Rate (Hz)
1	AAAAAAAAAAAAAA	1	5
2	ABCDEFGHIJKL	12	60
3	AAAAAAAABBBBBB	2	10
4	AAABBBAAABBB	2	20
5	ABABABABABAB	2	60
6	AAABBBCCCDDD	4	20
7	ABCDABCDABCD	4	60
8	AABBCCDDEEFF	6	30
9	ABCDEFABCDEF	6	60

## Data Availability

The script containing the complete statistical analyses is publicly available on the Open Science Framework (OSF) at https://osf.io/azynh/?view_only=a259e898898c4a6fa1bee3014defef5c (accessed on 30 July 2025). The dataset available on OSF includes only the data from Donato et al. [41], which are shared with the authors’ permission. Due to data sharing agreements, the dataset from the second study [40] is not publicly available. Researchers interested in accessing these data should contact the corresponding authors of Nankoo et al. [40] directly.

## Abbreviations

The following abbreviations are used in this manuscript:

GPs	Glass patterns
hMT+	Human middle temporal complex
LMM	Linear mixed-effects models
ANOVA	Analysis of variance
UML	Updated maximum-likelihood
QUEST	Quick estimation by sequential testing
OSF	Open Science Framework
LOC	Lateral occipital complex
STFI	Spatiotemporal form integration
EEG	Electroencephalogram
fMRI	Functional magnetic resonance imaging
MEG	Magnetoencephalography
